# Co_2_P Nanoparticles Wrapped in Amorphous Porous Carbon as an Efficient and Stable Catalyst for Water Oxidation

**DOI:** 10.3389/fchem.2018.00580

**Published:** 2018-11-22

**Authors:** Zunjian Ke, Haojie Wang, Dong He, Xianyin Song, Chongyang Tang, Jiangchao Liu, Lanli He, Xiangheng Xiao, Changzhong Jiang

**Affiliations:** ^1^Department of Physics and Key Laboratory of Artificial Micro- and Nano-structures of Ministry of Education, Hubei Nuclear Solid Physics Key Laboratory, Wuhan University, Wuhan, China; ^2^Key Laboratory of Materials Physics, Centre for Environmental and Energy Nanomaterials, Anhui Key Laboratory of Nanomaterials and Nanotechnology, Institute of Solid State Physics, Chinese Academy of Sciences, Hefei, China; ^3^Su Zhou Institute of Wuhan University, Suzhou, China

**Keywords:** water oxidation, Co_2_P, porous carbon, anchoring structure, charge transfer resistance

## Abstract

Exploring highly active, enduringly stable, and low-cost oxygen evolution reaction catalysts continues to be a dominant challenge to commercialize renewable electrochemical water-splitting technology. High-active and earth-abundant cobalt phosphides are recently considered as promising candidates. However, the poor inherent electron transfer efficiency and instability hinder its further development. In this work, a novel approach was demonstrated to effectively synthesize Co_2_P nanoparticles wrapped in amorphous porous carbon framework (Co_2_P/C). Benefiting from extremely high specific surface area of porous carbon, plenty of active sites were adequately exposed. Meanwhile, unique anchoring structure between Co_2_P nanoparticles and amorphous carbon outerwear insured high charge transfer efficiency and superior stability of Co_2_P/C. Due to these favorable properties, low overpotential of 281 mV at 10 mA cm^−2^ and Tafel slope of 69 mV dec^−1^ were achieved in resultant Co_2_P/C catalyst. More significantly, it only exhibited a negligible overpotential increase after 30 h stability test, and these performances entirely preceded commercial RuO_2_ benchmark. In summary, we proposed a simple and feasible strategy to prepare metal phosphides wrapped with amorphous porous carbon outerwear for efficient and durable electrochemical water oxidation.

## Introduction

Due to the rising global population, increasing energy demands, and deteriorating climate, it has become a significant research topic to develop renewable energy alternatives (Seh et al., 2017). One prospective goal is to develop electrochemical water splitting technology for converting water into oxygen (oxygen evolution reaction, OER) and hydrogen (hydrogen evolution reaction, HER) which is regarded as the most excellent fuel alternative for conventional fossil energy because of its high energy density and non-pollution (Roger et al., 2017; Seh et al., 2017). Compared to two-electron process of HER, OER process with four-electron transfer, the bottleneck in improving water-splitting technology, has aroused researchers' extensive attention in past several decades, in which even the most robust precious-metal-based catalyst (RuO_2_, IrO_2_) needs a substantial overpotential to realize the desired current density of 10 mA cm^−2^ at least (Zhang et al., 2016).

In recent years, plenty of transition-metal-based OER electrocatalysts have been reported because of their relatively high intrinsic activities and low cost, mainly including transition-metal oxides (Xu et al., 2016a), oxyhydroxides (Smith et al., 2013; Friebel et al., 2015), selenides (Xu et al., 2017), and phosphides (Xu et al., 2016b; Song et al., 2017; Ye et al., 2017; Hong et al., 2018; Zhang et al., 2018). Among these extensively researched electrocatalysts, transitional metal phosphides have attracted considerable attention (Gao et al., 2018). Typically, *Hu's* group first reported that the evolution of core-shell (Ni_2_P/NiO_x_) structure in Ni_2_P enhanced its OER performance (Stern and Hu, 2016). *Lin's* group reported a facile sugar-blowing technique and low-temperature phosphorization to generate 3D self-supported metal involved carbon nanostructures termed as Co_2_P@Co/nitrogen doped carbon for catalyzing OER (Zhu et al., 2017). *Justus* et al presented a simple and safe method to prepare cobalt-cobalt phosphide (Co/Co_2_P) nanoparticles under reductive conditions for stably driving basic oxygen evolution reaction (Masa et al., 2016). Although a spate of recent research progress about the cobalt phosphides were reported, their properties were still unsatisfying, mainly due to the higher overpotential and poor catalytic stability during OER process. This was mainly ascribed to its semi-conductive properties, sharply hindering electron transfer in OER process (Wang et al., 2017).

To date, some feasible and effective approaches to improve conductivity and catalytic performance of cobalt phosphides include fabricating multi-metallic catalyst by taking advantage of synergistic metal-metal interaction (Chen G. F. et al., 2016; Liang et al., 2016; Li et al., 2016; Song et al., 2017) and fabricating porous nanostructures (You et al., 2015; Feng et al., 2016; Tan et al., 2016). Introducing carbon materials as favorable supports was also occasionally reported to accelerate the charge transfer; however, it was not powerful to make catalyst maintain stable in a long-term water oxidation process (Zhu et al., 2017). Consequently, the strategy to give consideration to both charge transfer and stability is vital for further improvement of cobalt phosphides.

In this work, we demonstrated a novel, low-cost and scalable approach to synthesize Co_2_P nanoparticles uniformly embedded in amorphous porous carbon support (Co_2_P/C). The prepared Co_2_P/C presented a distinct encapsulation structure, in which Co_2_P nanoparticles were toughly wrapped by amorphous carbon outerwear and uniformly distributed in this amorphous porous carbon. This favorable structural feature insured the exposure of more active sites on carbon framework, sharply decreased the charge transfer resistance and improved catalytic stability. Consequently, excellent electrochemical properties were realized. To be specific, the result Co_2_P/C merely needs a low overpotential of 281 mV to reach the current density of 10 mA cm^−2^, under 291 mV for RuO_2_. Remarkably, a low Tafel slope of 69 mV dec^−1^ for Co_2_P/C verified its superior activity in comparison with RuO_2_ benchmark (160 mV dec^−1^). What's more, a negligible overpotential increase after 30 h stability test indicated superior electrocatalytic stability.

## Results and discussion

**Figure 1:**
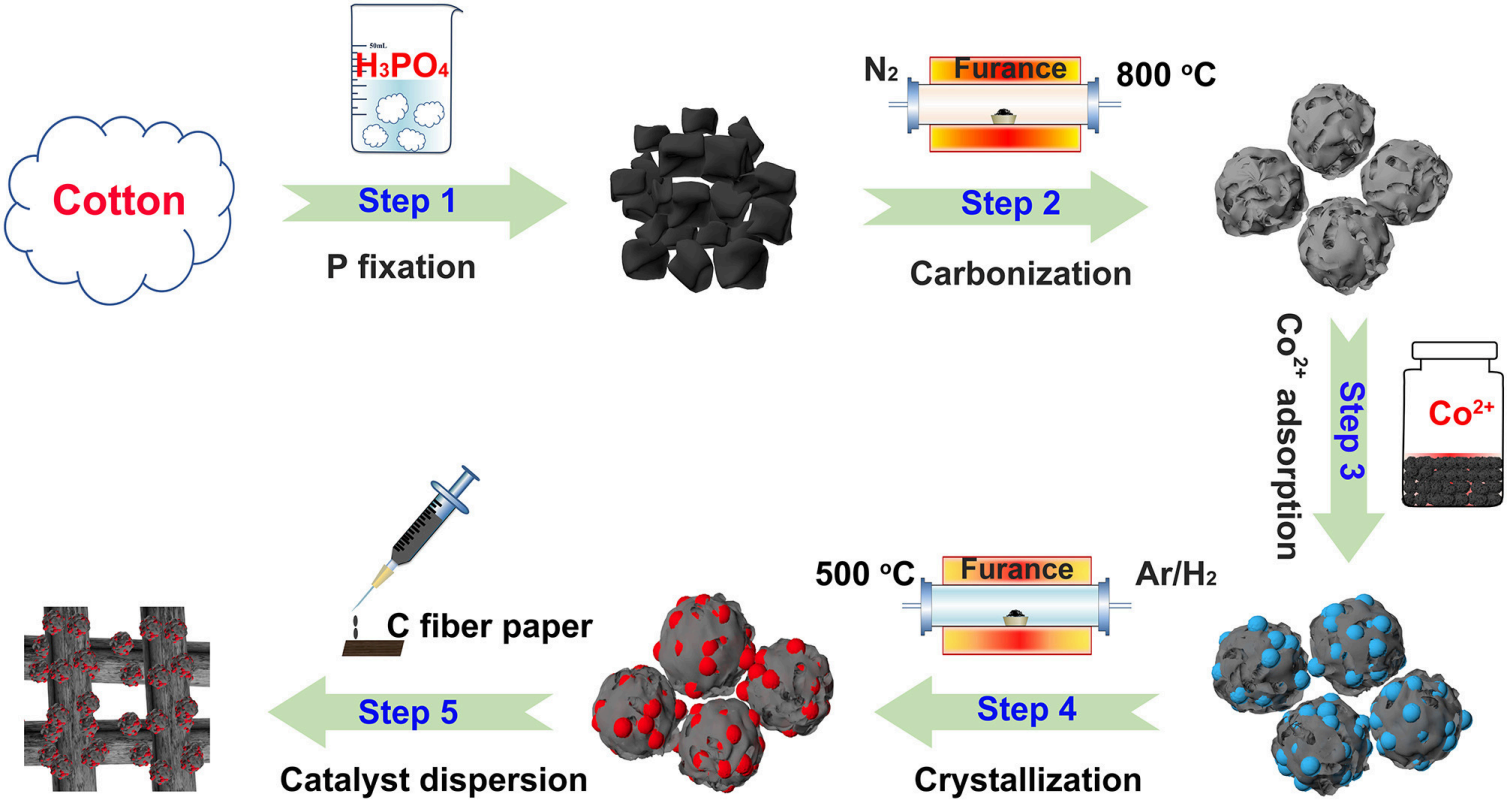
Schematic diagram of process to prepare Co_2_P/C catalyst.

**Figure 2:**
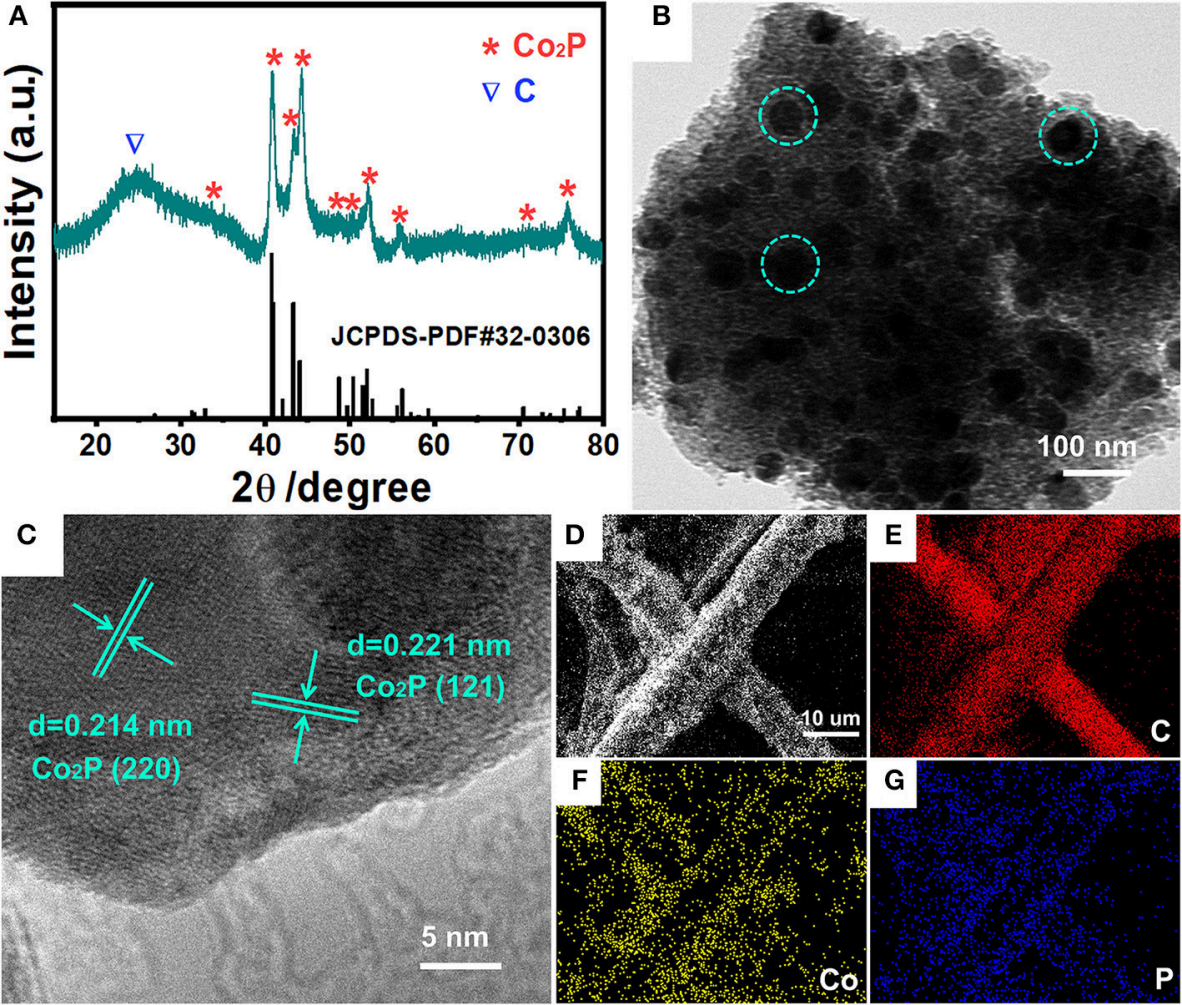
Structure and composition characterizations of Co_2_P/C. **(A)** XRD patterns, **(B)** TEM image, **(C)** HRTEM image, **(D)** SEM image and the corresponding elemental mapping images of C **(E)**, Co **(F)**, P **(G)** for Co_2_P/C.

A novel, low-cost and scalable approach was demonstrated to synthesize Co_2_P nanoparticles uniformly embedded in porous carbon support, as illustrated visually in Figure 1. Typically, we adopt phosphoric acid and cotton as phosphorous and carbon source of Co_2_P/C catalyst, respectively. P was fixed in porous carbon after a carbonization process in N_2_. Thus, by means of Co^2+^ adsorption and subsequently annealing in Ar/H_2_, the Co_2_P nanoparticles uniformly wrapped in porous carbon structure were obtained. The fabricated catalyst was firstly revealed the crystalline composition via X-ray diffraction (XRD) analysis, as shown in Figure 2A. The sharp diffraction peaks at 32.9, 40.7, 40.9, 43.3, 44.0, 48.7, 50.4, 52.0, 57.2, 71.0, and 75.3° can be indexed to the crystal planes of (111), (121), (201), (211), (130), (031), (310), (002), (311), (132), and (312) of Co_2_P (JCPDS no. 32-0306; Dutta et al., 2016; Masa et al., 2016; Das et al., 2017), respectively, firmly confirming that Co_2_P nanoparticles was successfully prepared by our method. The wide diffraction peak located at ~26.4° was considered to derive from the carbon support (Wang et al., 2017).

A transmission electron microscopy (TEM) image was recorded to illustrate the morphology and distribution of Co_2_P/C catalyst, as displayed in Figure 2B. Obviously, Co_2_P exhibited the morphology of nanoparticles with uniform sizes, and these nanoparticles (green dashed circle in Figure 2B) were equably embedded in carbon support. To further confirm black spots in Figure 2B were indeed Co_2_P nanoparticles, the detailed structural information of these black spots were demonstrated by high-resolution transmission electron microscopy (HRTEM) image shown in Figure 2C. The lattice distance of 0.205 and 0.221 nm corresponds to the (130) and (121) plane of Co_2_P, respectively, strongly demonstrating that Co_2_P nanoparticles were successfully obtained. Meanwhile, distinct interface between the Co_2_P nanoparticle and amorphous carbon layer (Figures S1A,B) further suggested the Co_2_P nanoparticle was tightly wrapped in amorphous carbon layer. More importantly, the amorphous carbon layer displayed a uniform porous morphology (Figure S1C). These results convincingly reveal that Co_2_P nanoparticles are successfully synthesized and anchored in amorphous porous carbon support. In addition, field-emission scanning electron microscopy (FESEM) image and corresponding element mapping of C, Co, and P for Co_2_P/C catalyst loaded on carbon fiber paper were displayed in Figures 2D–G. The distinct carbon fiber profile presented in C, Co, and P mapping indicated that Co_2_P/C catalyst was uniformly coated on carbon fiber paper, which can be also verified by low-magnification SEM image and EDS spectra given in Figures S2A,B. These results further confirmed the successful formation of Co_2_P nanoparticles and their uniform distribution in amorphous porous carbon support.

**Figure 3:**
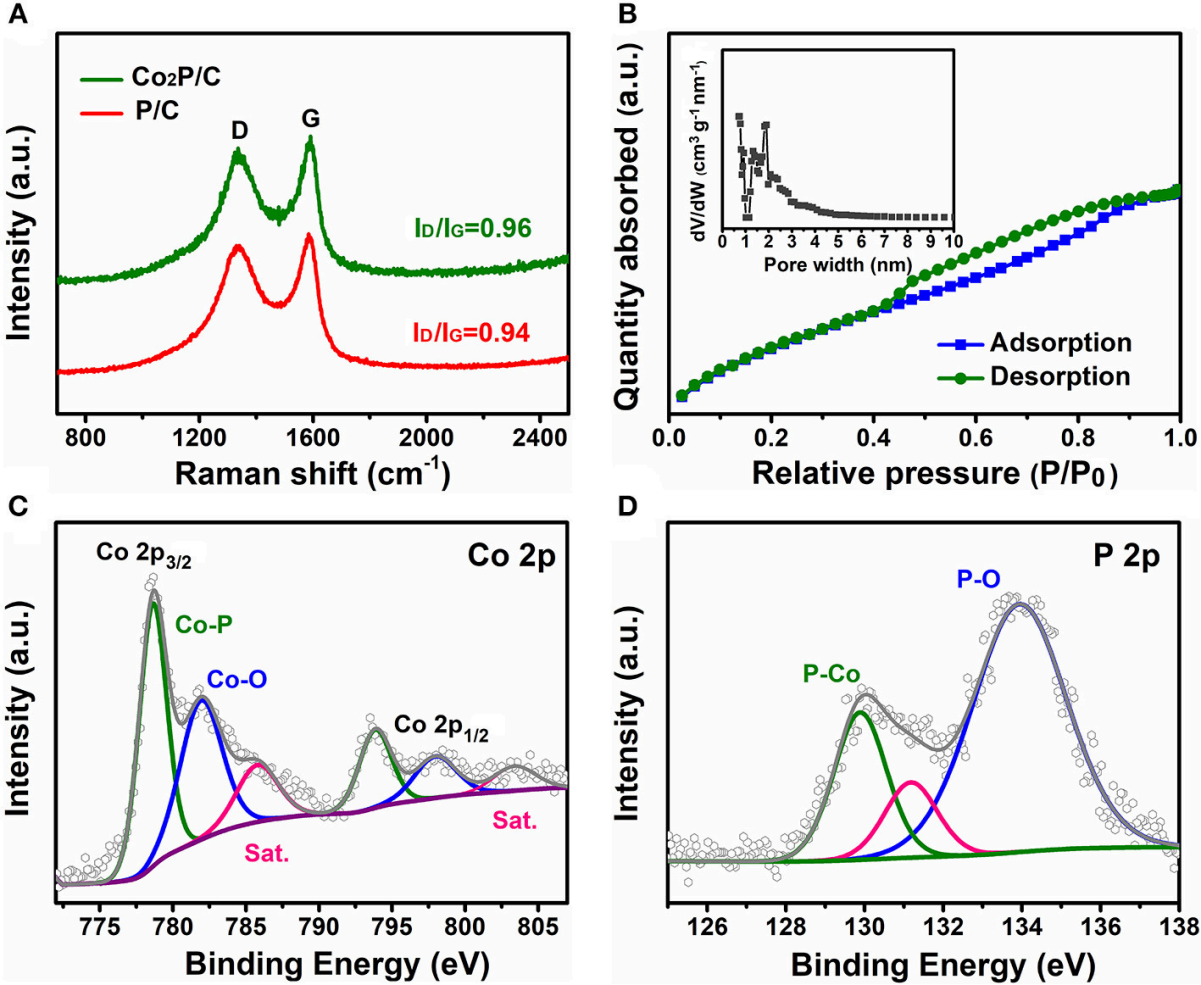
**(A)**Raman spectra, **(B)** N_2_ sorption isotherms (the inset shows the corresponding pore size distribution) and high-resolution XPS spectra of Co_2_P/C: Co 2p **(C)** and P 2p **(D)**.

To research the structural and compositional changes of carbon support induced by the introduction of Co, Raman spectroscopy was employed (Figure 3A). Apparently, Co_2_P/C and P/C samples (without Co species adsorption) both displayed two explicit characteristic D and G bands located at 1,350 cm^−1^ and 1,587 cm^−1^, which were associated with defects and graphitization (Xia et al., 2016; Wang et al., 2017). Compared with the P/C sample, Co_2_P/C exhibited a higher value of I_D_/I_G_, indicating a higher degree of graphitization in Co_2_P/C samples (Wang et al., 2017; Zhang et al., 2018). This could be ascribed to the catalysis of Co nanoparticles derived from Co^2+^ reduction in Ar/H_2_ annealing process (Wang et al., 2017). N_2_ sorption isotherms of the prepared Co_2_P/C powder were measured to evaluate the textural porosity and surface areas of carbon framework (Figure 3B). The isotherm with a remarkable hysteresis loop for the Co_2_P/C sample suggested a mesoporous structure (Xia et al., 2016; Wang et al., 2017; Zhu et al., 2017), well corresponding with the result in Figure S1C. And the Co_2_P/C sample presented a much high BET surface area of 869.697 m^2^/g. This high surface area with an average pore size of 1.92 nm for the Co_2_P/C sample is anticipated to expose more active sites, increase the electrochemical active areas and accelerate proton transfer.

X-ray photoelectron spectroscopy was used to investigate the surface elemental compositions and chemical states of Co_2_P/C. The survey spectrum in Figure S3A indicated the presence of C, O, Co, and P species, and no other elements were observed. Obviously, the peaks of C, Co and P were derived from the Co_2_P/C sample, and the appearance of O element was considered to be caused by adsorption of oxygen or H_2_O in the air (Wang et al., 2017). The corrected (C 1s, Figure S3B) high-resolution Co 2p and P 2p spectra are illustrated in Figures 3C,D. The peaks located at 778.6 and 129.9 eV demonstrated that the presence of Co-P bond (Zhang et al., 2017; Zhu et al., 2017; Thalluri et al., 2018). Meanwhile, the peaks observed at 781.9 and 798.0 eV in the Co 2p spectra, both with obvious satellite peaks, are assigned to the oxidized Co species. Similarly, an oxidized P species peak was also observed at 133.9 eV in the P 2p (Figure 3D; Chen P. et al., 2016; Masa et al., 2016; Zhang et al., 2017; Zhu et al., 2017; Wang et al., 2018).

**Figure 4:**
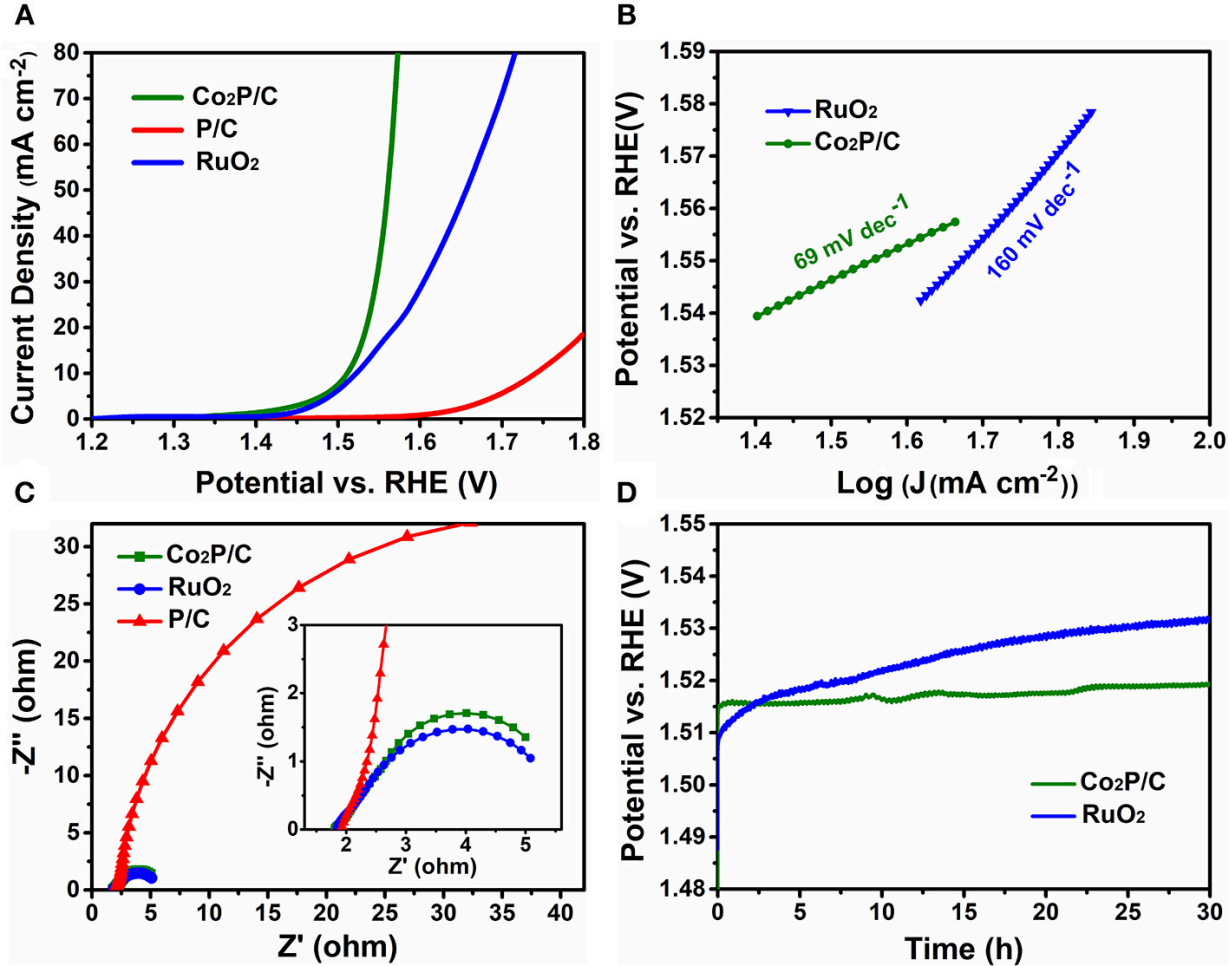
Electrochemical OER performance in 1.0 M KOH solution. **(A)** IR-corrected (95%) LSV polarization curves at a scan rate of 5 mV/s, **(B)** Tafel slope plots, **(C)** Nyquist plots of electrochemical impedance spectra (the inset is a high-magnification presentation) at η_overpotential_ = 300 mV (vs. RHE), and **(D)** Time-dependent potential curves under a static current density of 10 mA cm^−2^.

IR-corrected linear sweep voltammetry (LSV) measurements were first recorded in 1.0 M KOH solution to evaluate the electrocatalytic properties of porous Co_2_P/C in comparison with P/C and commercial RuO_2_ (Figure 4A). Obviously, the IR-corrected LSV curve of porous P/C sample shows an sluggish oxygen evolution process, while the Co_2_P/C exhibits a swift OER response, indicating that the introduction of Co-based active sites in form of cobalt phosphide played a dominate role in promoting the electrocatalytic performance. Remarkably, thanks to the improved charge transfer efficiency of the encapsulation structure, the resultant Co_2_P/C achieves a current density of 10 mA cm^−2^ at a low over potential of 281 mV, which even precedes 291 mV of commercial RuO_2_ benchmark. What's more, with the potential in Faradic range increasing, the current density of Co_2_P/C rises more quickly than RuO_2_, manifesting a faster catalysis kinetics of Co_2_P/C than RuO_2_, and this can be proved accordingly by Tafel plots presented in Figure 4B. The Co_2_P/C sample displayed a Tafel slope of 69 mV dec^−1^, which was much lower than that of RuO_2_ (160 mV dec^−1^), further revealing the excellent oxygen evolution reaction kinetics of the prepared porous Co_2_P/C catalyst.

Moreover, the charge transfer resistance was another vital parameter to illustrate electrochemical properties, which can be gained from electrochemical impedance spectra (Figure 4C). The arc diameter of the Nyquist plot reflected the value of transfer resistance. It was clear that porous Co_2_P/C sample showed a lower transfer resistance than P/C sample, indicating that introduction of Co caused partially graphitization of porous carbon support, which sharply decreased charge transfer resistance and improved the OER performance of Co_2_P/C. Besides, a similar transfer resistance of Co_2_P/C in comparison with the value of RuO_2_ also verifies that Co_2_P/C has comparable electrocatalytic properties relative to RuO_2_. Electrochemical double-layer capacitance (C_dl_) was tested to address the effect of porous carbon structure (Figure S4A). A C_dl_ value of 127.4 mF cm^−2^ which was higher than other carbon based cobalt phosphides (Zhu et al., 2017; Yang et al., 2018) indicated a large electrochemical active area of Co_2_P/C. And this could be ascribed to the porous structure of support in Co_2_P/C catalyst, which exposed more active sites and thus increased active areas. These analysis was well corresponding to results in Figure 3B and Figure S1C.

To assess the electrocatalytic stability of porous Co_2_P/C catalyst, the 30 h potential-time (V-T) curves of Co_2_P/C and RuO_2_ electrodes at a fixed current density of 10 mA cm^−2^ were presented in Figure 4D. Apparently, due to a constantly decayed activity in basic solution, the RuO_2_ electrode had a well-marked overpotential increase after 30 h V-T test; however, the Co_2_P/C exhibited a negligible overpotential rise (only ~5 mV) after the same durability test, displaying its superior electrochemical stability. These significant properties can be attributed to the novel and favorable structure of Co_2_P/C catalyst, in which the Co_2_P nanoparticle was strongly and uniformly anchored in amorphous porous carbon (Figure 2B, Figure S1B). This structural features insured active sites were not easy to aggregate and peel off during OER process. Accordingly, the fact that there was no obvious distinction in the LSV plots of Co_2_P/C electrode before and after 30 h durability test (Figure S4B) further confirmed the Co_2_P/C catalyst indeed exhibited superior stability in alkaline medium.

## Conclusion

In summary, this work developed Co_2_P nanoparticles uniformly wrapped in amorphous porous carbon support as an efficient and robust electrocatalyst to drive oxygen evolution reaction via phosphorylation of cotton and subsequently carbonization and reduction process after introducing Co species. Benefiting from the much high specific surface area of porous support for Co_2_P/C catalyst, plenty of exposed active sites and increased electrochemical active areas were achieved. Meanwhile, thanks to the favorable structure of Co_2_P/C catalyst, in which the Co_2_P nanoparticle was strongly and uniformly anchored in amorphous carbon, the charge transfer resistance of Co_2_P was sharply decreased, and more importantly, the exposed active sites in amorphous carbon were hard to peel off. These significant properties derived from unique advantageous structure resulted in a superior electrocatalytic OER performance. The prepared Co_2_P/C catalyst achieved a current density of 10 mA cm^−2^ at a lower overpotential of 281 mV than RuO_2_ (291 mV). Besides, Tafel plots indicated the Co_2_P/C sample had a faster oxygen evolution reaction kinetics in comparison with RuO_2_. More importantly, in contrast to the obvious overpotential increase of commercial RuO_2_, the Co_2_P/C sample almost maintained initial superior OER activity after a 30 h stability test. Apart from providing a promising electrocatalyst alternative, this work proposed a simple and cost-effective strategy to prepare metal phosphide encapsulated with porous carbon materials electrocatalyst for electrochemical energy conversion and storage.

## Experimental section

### Preparation

To be specific, 1.0 g degreasing cotton was immersed into 32 ml deionized water dissolving 2 ml phosphoric acid. After an ultrasonic treatment for 5 min, the beaker was moved into air oven and dried in 100 120°C. Subsequently, the attained black blocks were placed in the tube furnace and annealed at 800°C in N_2_ for 3 h. After naturally cooling to room temperature and a vigorously grinding, the resultant powder was soaked in 0.5 ml 3 M Co(NO_3_)_2_.6H_2_O solution and then strongly dispersed by an ultrasonic treatment to make Co^2+^ adsorb adequately and tightly. After that, the mixture was moved to air oven and dried in 60 80°C. Adjacently, the completely dried powder was transferred into tube furnace and annealed at 500°C 650°C for 2 h in Ar/H_2_ mixture gas (10%). After naturally cooling to room temperature and a strongly grinding, the Co_2_P catalyst uniformly wrapped in porous carbon support (Co_2_P/C) was obtained.

### Characterizations

X-ray diffraction (XRD) was carried out on Bruker AXS, D8 Advance X-ray powder diffractometer with Cu-Kα radiation (λ = 0.15418 nm, Germany). High-resolution transmission electronic microscope (HRTEM) was performed by JEOL 2010 (HT) operating at 200 kV. Scanning electron microscopy (SEM) and energy dispersive X-ray spectroscopy (EDS) were carried out using a FEI Sirion FEG scanning electron microscope at accelerating voltage of 20 kV. Raman measurements were performed by laser confocal micro Raman spectrometers (Renishaw in Via, Renishaw, UK) with laser excited at 532 nm. X-ray photoelectron spectroscopy (XPS) measurements were done on a Thermo Scientific ESCALAB 250 Xi system (USA) with Al Kα (1486.6 eV) as the radiation source and all spectra were calibrated using the C 1s peak with a value of 284.6 eV.

### Electrochemical measurements

To characterize electrochemical performance of Co_2_P/C catalyst, 2.5 mg Co_2_P/C powder was dispersed into 0.25 mL isopropanol dissolving 5 uL nafion solution. After an ultrasonic concussion for 1 h, the mixed liquid was drip-coated on carbon fiber paper with a micropipette. Finally, the electrode sample were placed on heating stage and dried at 60°C for 1 h before performance measurements. For comparison with Co_2_P/C sample, 2.5 mg RuO_2_ and integrated P/C powder were also dispersed and coated on carbon fiber paper according to the same process. All electrochemical measurements were performed in an undivided three-electrode cell using an electrochemical workstation (Chenhua CHI 660D). A Pt foil and Ag/AgCl (saturated KCl) were used to record potential as the counter and reference electrode, respectively. The potential vs. the reference electrode was converted to the potential vs. reversible hydrogen electrode (RHE) by the equation below:

$$\begin{array}{r} E\left(RHE\right)=E\left(Ag/AgCl\right)+\;0.059\;pH+0.197 \end{array}$$

Electrochemical measurements were taken in 40 ml 1.0 M KOH solution. Practical effective areas of all samples were 1 cm^2^.

## Author contributions

XX, CJ, and ZK designed and finished the synthesis and characterization analysis of materials and wrote the research paper. HW, DH, XS, CT, JL, and LH offered many valuable suggestions to materials preparation, data processing, and paper modification.

### Conflict of interest statement

The authors declare that the research was conducted in the absence of any commercial or financial relationships that could be construed as a potential conflict of interest.
