# Supplementary material for: Co2P Nanoparticles Wrapped in Amorphous Porous Carbon as an Efficient and Stable Catalyst for Water Oxidation
Source: Front Chem. 2018 Nov 22;6:580. doi: 10.3389/fchem.2018.00580 (PMC6262043; doi:10.3389/fchem.2018.00580)
Supplement: Supplementary file 1 [file Table_1.DOCX]

Supplementary Materials

**Co_2_P Nanoparticles Wrapped in Amorphous Porous Carbon as An Efficient and Stable Catalyst for Water Oxidation**

Zunjian Ke^1^, Haojie Wang^2^, Dong He^1^, Xianyin Song^1^, Chongyang Tang^1^, Jiangchao Liu^1^, Lanli He^1^, Xiangheng Xiao^1*^, Changzhong Jiang^1^

^1^ Department of Physics and Key Laboratory of Artificial Micro- and Nano-structures of Ministry of Education, Hubei Nuclear Solid Physics Key Laboratory, Wuhan University, Wuhan 430072, China

^2^ Key Laboratory of Materials Physics, Centre for Environmental and Energy Nanomaterials, Anhui Key Laboratory of Nanomaterials and Nanotechnology, Institute of Solid State Physics, Chinese Academy of Sciences, Hefei 230031, Anhui, China

# Supplementary Figures


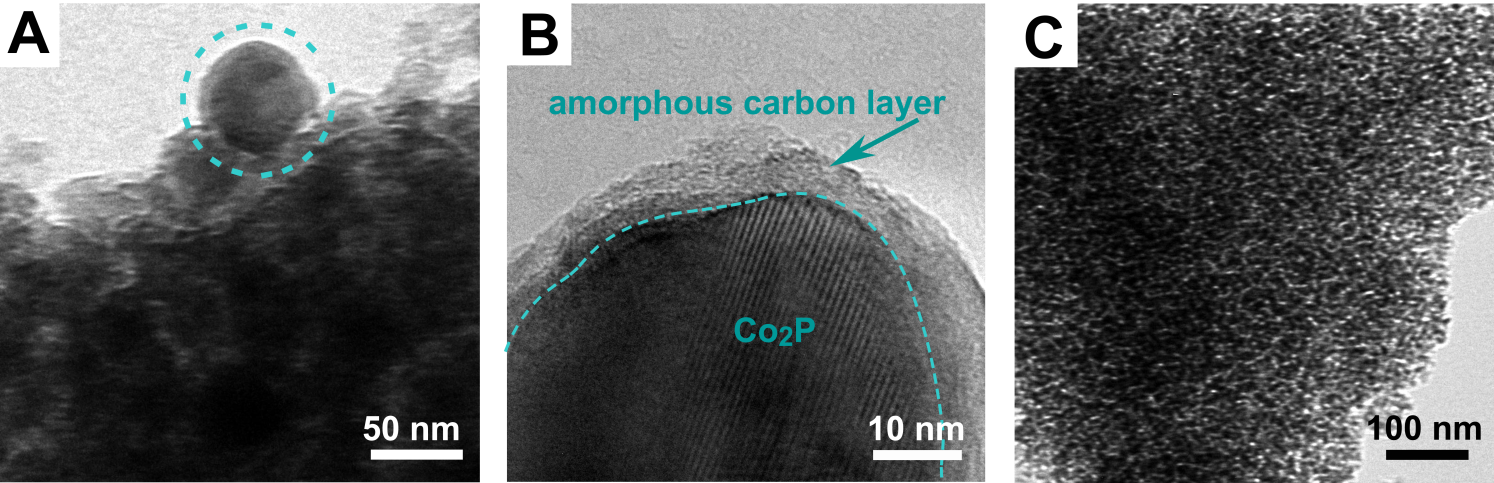


**Supplementary Figure 1.** (**A**) TEM and (**B**) HRTEM of Co_2_P/C. (**C**) TEM of P/C samples without Co species.

**
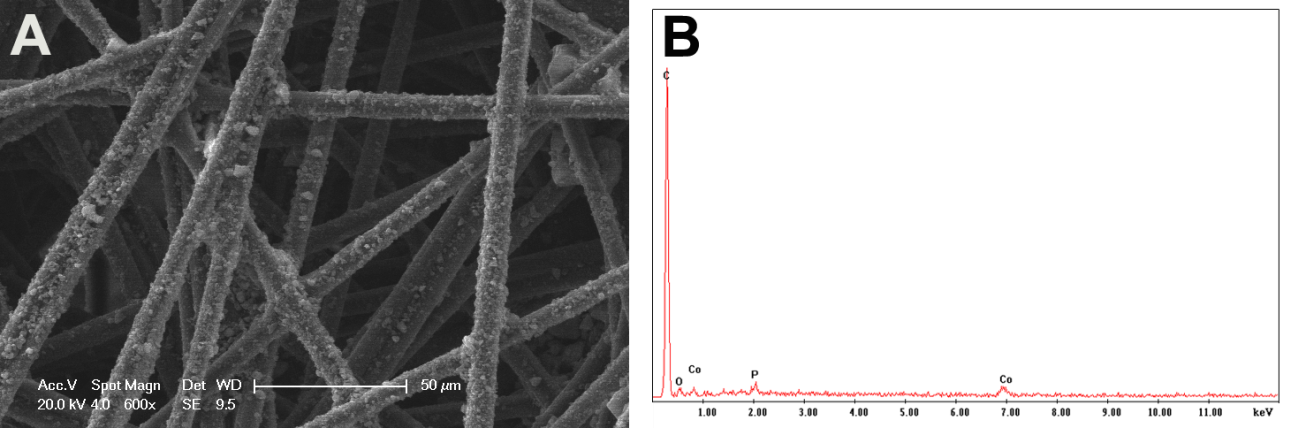
**

**Supplementary Figure 2.** (**A**) SEM image of Co_2_P/C sample on Carbon fiber paper. (**B**) EDS spectrum for Co_2_P/C powder.


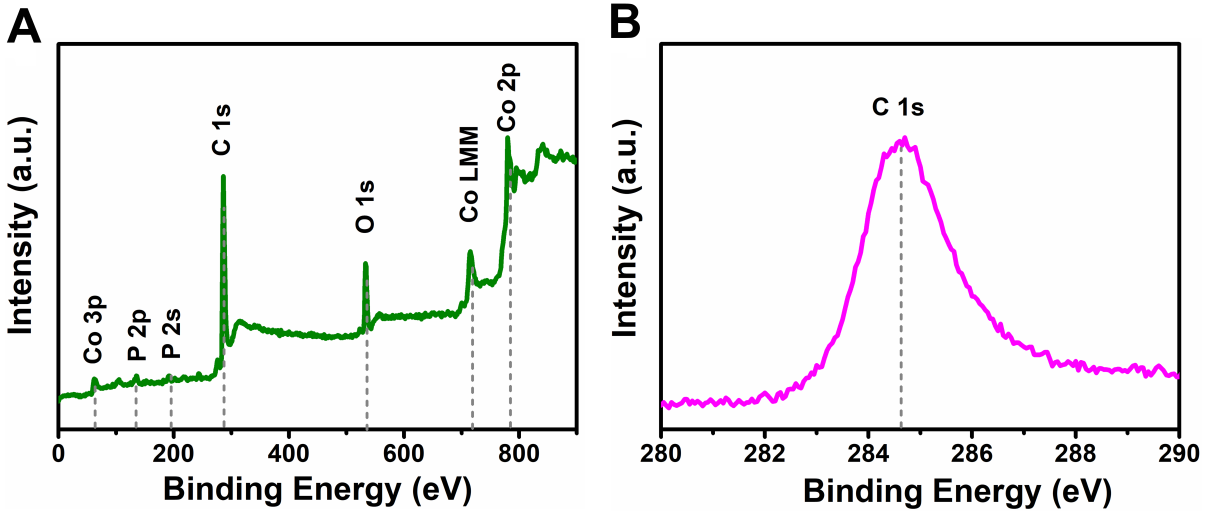


**Supplementary Figure 3.** XPS spectra. (**A**) The survey spectrum. (**B**) the C 1s fine spectrum.

**
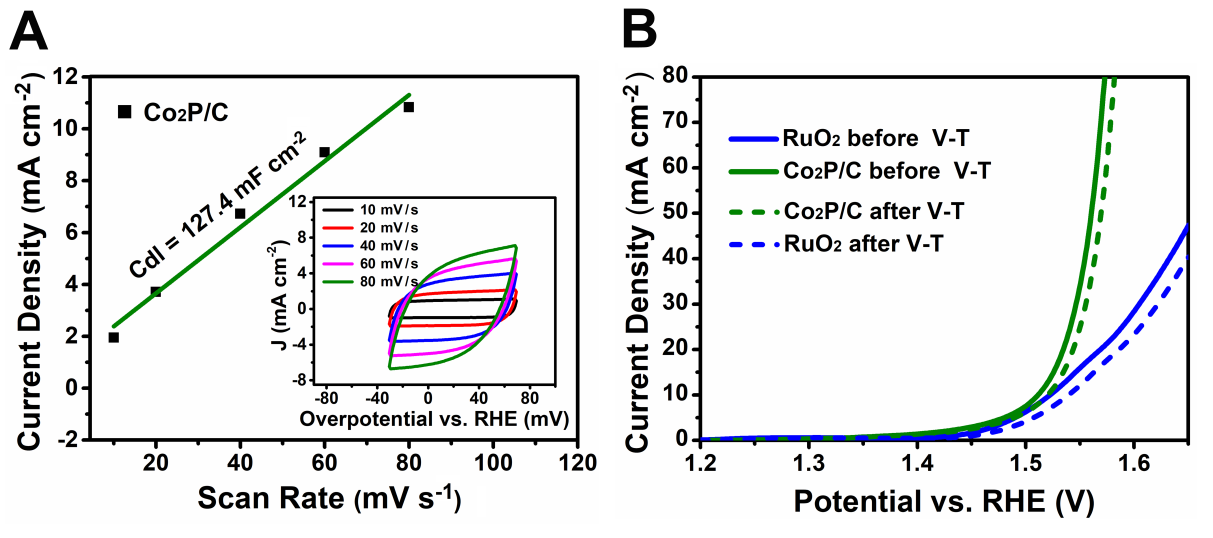
**

**Supplementary Figure 4.** (**A**) Scan rate-independent current density of Co_2_P/C at 1.03 V versus RHE, and the C_dl_ of electrocatalyst was determined from the slope of fitted line (the inset was CV plots at scan rates between 10 and 80 mV /s). (**B**) IR-corrected LSV polarization curves for Co_2_P/C and commercial RuO_2_ before and after 30 hours durability test.
